# Genetic, clinical and imaging implications of a noncompaction phenotype population with preserved ejection fraction

**DOI:** 10.3389/fcvm.2024.1337378

**Published:** 2024-02-06

**Authors:** Kinga Grebur, Balázs Mester, Bálint András Fekete, Anna Réka Kiss, Zsófia Gregor, Márton Horváth, Kristóf Farkas-Sütő, Katalin Csonka, Csaba Bödör, Béla Merkely, Hajnalka Vágó, Andrea Szűcs

**Affiliations:** ^1^ Heart and Vascular Center, Semmelweis University, Budapest, Hungary; ^2^ Department of Pathology and Experimental Cancer Research, Semmelweis University, Budapest, Hungary

**Keywords:** hypertrabeculation, noncompaction, excessive trabeculation, cardiogenetics, risk stratification, cardiac magnetic resonance imaging

## Abstract

**Introduction:**

The genotype of symptomatic left ventricular noncompaction phenotype (LVNC) subjects with preserved left ventricular ejection fraction (LVEF) and its effect on clinical presentation are less well studied. We aimed to characterize the genetic, cardiac magnetic resonance (CMR) and clinical background, and genotype-phenotype relationship in LVNC with preserved LVEF.

**Methods:**

We included 54 symptomatic LVNC individuals (LVEF: 65 ± 5%) whose samples were analyzed with a 174-gene next-generation sequencing panel and 54 control (C) subjects. The results were evaluated using the criteria of the American College of Medical Genetics and Genomics. Medical data suggesting a higher risk of cardiovascular complications were considered “red flags”.

**Results:**

Of the LVNC population, 24% carried pathogenic or likely pathogenic (P) mutations; 56% carried variants of uncertain significance (VUS); and 20% were free from cardiomyopathy-related mutations. Regarding the CMR parameters, the LVNC and C groups differed significantly, while the three genetic subgroups were comparable. We found a significant relationship between red flags and genotype; furthermore, the number of red flags in a single subject differed significantly among the genetic subgroups (*p* = 0.002) and correlated with the genotype (*r* = 0.457, *p* = 0.01). In 6 out of 7 LVNC subjects diagnosed in childhood, P or VUS mutations were found.

**Discussion:**

The large number of P mutations and the association between red flags and genotype underline the importance of genetic-assisted risk stratification in symptomatic LVNC with preserved LVEF.

## 1 Introduction

Left ventricular hypertrabeculation or left ventricular noncompaction (LVNC) has recently become the subject of renewed interest, as novel morphogenetic studies have modified the theory of trabecular and compact myocardial development (1, 2).

**Figure 1:**
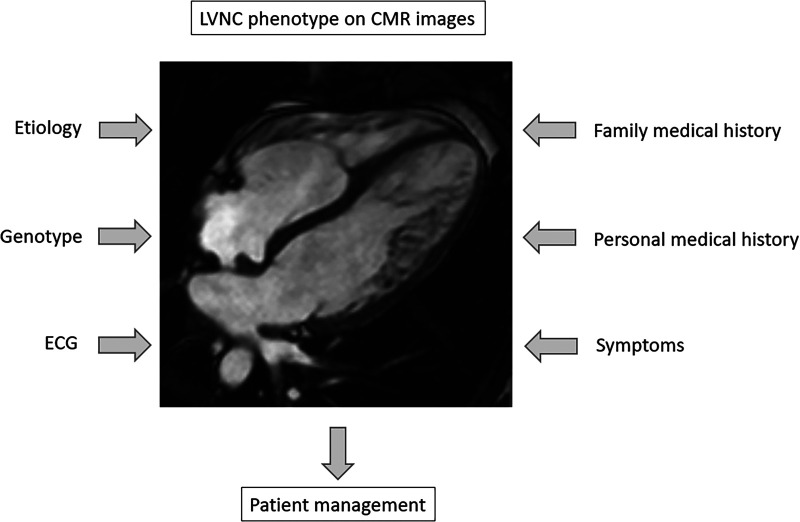
Diagnostic aspects in the assessment of left ventricular hypertrabeculation. CMR, cardiac magnetic resonance imaging, ECG, electrocardiogram; LVNC, left ventricular noncompaction.

The gold standard diagnostic tool to assess the LVNC phenotype is cardiac magnetic resonance imaging (CMR), which provides different criteria to evaluate this phenomenon (3–8). Despite the same phenotypic trait, the spectrum of LVNC could be wide, ranging from symptomless healthy individuals through LVNC with minor symptoms to LVNC with cardiomyopathy (CMP), systolic dysfunction or severe complications (1, 3, 7, 9–11). Thus, medical management should be based on clinical characteristics: previous personal and family medical history, electrocardiogram (ECG) records and clinical symptoms of heart failure (HF), ventricular arrhythmias and thromboembolic events (1, 9, 12–14). New evidence also suggests the importance of determining the underlying etiology to distinguish acquired, transient morphology, e.g., sport adaptation and pregnancy, from the genetic forms (2, 12, 15–17). Genetic studies describing sarcomeric genes, e.g., TTN, TNNT2, MYH7 and MYBPC3 in association with “excessive trabeculation” suggest the importance of genetic analyses in symptomatic LVNC (Figure 1) (13, 18–20).

Although several articles conducted mainly on LVNC subjects with HF describe high rates of genetic involvement, little is known about the genetic background and clinical manifestation of symptomatic LVNC with preserved left ventricular ejection fraction (LVEF) (1, 2, 18, 19, 21–23). Thus, it is an interesting, open question to investigate the genetic characteristics of this specific LVNC phenotypic group.

We aimed to describe the genetic and clinical characteristics of a symptomatic LVNC phenotypic population with preserved LVEF to assess the relationship of the genotype with CMR parameters and clinical manifestation and to compare the CMR parameters with a healthy control (C) group.

## 2 Materials and methods

### 2.1 Study population

In our cross-sectional study, we included 54 (33 male) symptomatic subjects with LVNC phenotype and preserved LVEF (≥50%) and 54 (33 male) sex- and age-matched C individuals from a Caucasian population. The baseline characteristics are reported in Table 1.

**Table 1:** The baseline characteristics of the study populations.

	Control group	Total LVNC population	*p*	Genetic subgroups			
				Benign	VUS	Pathogenic	*p*
*n* (male)	54 (33)	54 (33)	1.000	11 (7)	30 (20)	13 (6)	0.488
Age (years)	38 ± 14	39 ± 14	0.743	42 ± 15	39 ± 13	36 ± 16	0.592
LVEF (%)	69 ± 5	65 ± 5	<0.01*	66 ± 3	64 ± 6	65 ± 5	0.758
BMI (kg/m^2^)	24.3 ± 3.0	25.2 ± 4.2	0.148	25.3 ± 4.3	26.1 ± 4.4	23.4 ± 3.1	0.157
BSA (m^2^)	1.9 ± 0.2	2.0 ± 0.2	0.183	2.0 ± 0.2	2.0 ± 0.2	1.8 ± 0.2	0.158

BMI, body mass index; BSA, body surface area; LVEF, left ventricular ejection fraction; LVNC, left ventricular noncompaction; VUS, variant of uncertain significance; *p*, significance level.

* *p* < 0.05, mean ± standard deviation.

In the LVNC group, symptomatic subjects with a persistent isolated hypertrabeculated phenotype fulfilling the Petersen (noncompact/compact myocardial layer ≥2.3) and Jacquier LVNC criteria (noncompact myocardial mass >20% of the total myocardial mass) and preserved LVEF (≥50%) were enrolled at our expert CMR center (4, 5). Patients with reduced LVEF (<50%); congenital, ischemic or valvular heart diseases; other or overlapping CMP; hypertension; relevant comorbidities (see details at 2.4 Clinical evaluation); exercise activity of >6 h/week or other cause of transient hypertrabeculation were excluded from our study (24). Other exclusion criteria included technical reasons, e.g., artifacts, implanted devices or contrast agent administration before segmentation (25).

The C group included healthy volunteers without known cardiovascular or extracardiac diseases and with exercise activity of <6 h/week (24).

All procedures performed in this study were in accordance with the 1964 Helsinki Declaration and its later amendments or comparable ethical standards. Ethical approval was obtained from the Central Ethics Committee of Hungary, and all participants provided informed consent.

### 2.2 Image acquisition

The CMR examinations were performed using 1.5 T magnetic resonance imaging (MRI) scanners (Magnetom Aera, Siemens Healthineers, Erlangen, Germany and Achieva, Philips Medical System, Eindhoven, the Netherlands). Retrospectively gated, balanced steady-state free precession (bSSFP) cine sequences were performed with short-axis (SA) and two-, three-, and four-chamber long-axis views from base to apex, covering the whole left and right ventricle (LV and RV). The slice thickness was 8 mm with no interslice gap, and the field of view was 350 mm on average, adapted to body size.

Contrast agent was administered when it was needed to 50 LVNC subjects: 44 received gadobutrol (0.1 ml/kg), 5 received gadobenate (0.2 ml/kg) and 1 received gadoterate (0.2 ml/kg). In four LVNC subjects [3 from the variant of uncertain significance (VUS) group and 1 from the benign (B) group] and healthy controls, contrast agent was not administered. To provide the best image quality for postprocessing analyses, contrast agent administration occurred after the acquisition of SA cine images (25).

### 2.3 Image analysis

For postprocessing analysis, we used Medis Suite software (Medis Suite QMass, version 4.0, Medis Medical Imaging Systems, Leiden, the Netherlands).

**Figure 2:**
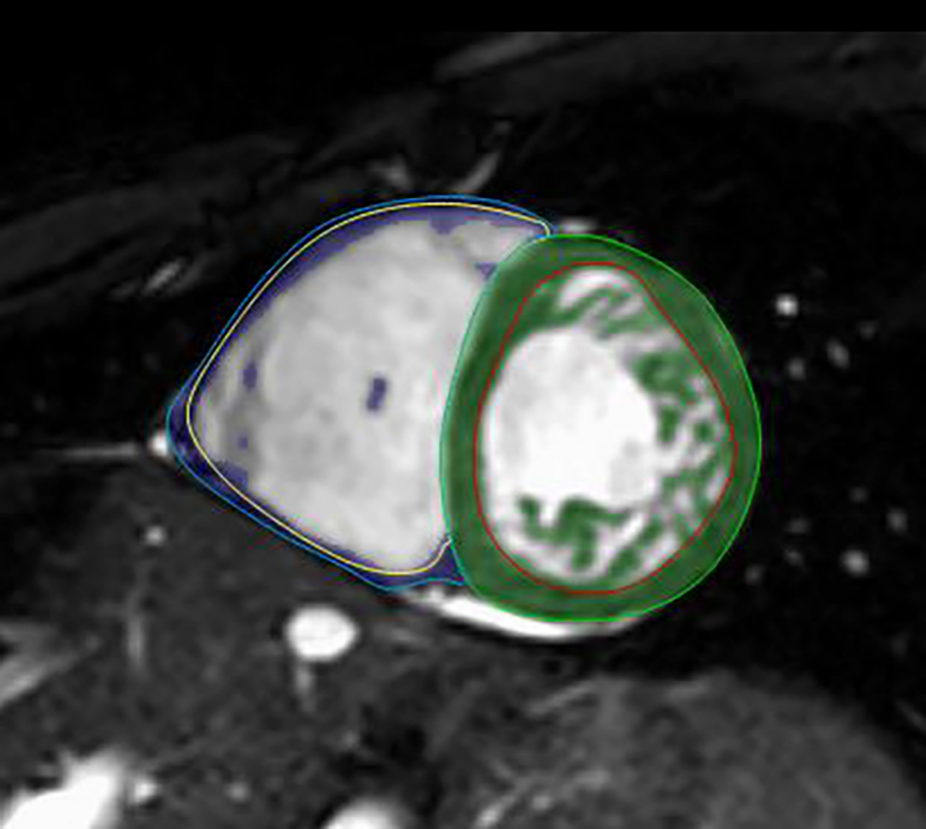
LVNC on short-axis CMR images with the threshold-based postprocessing method. Within the green (LV) and blue (RV) epicardial contours, the program identifies each voxel as blood or myocardium. The voxels identified as myocardium within the red (LV) and yellow (RV) endocardial borders represent the TPMi, which is green in the LV and purple in the RV. CMR, cardiac magnetic resonance imaging; LV, left ventricle; LVNC, left ventricular noncompaction; RV, right ventricle; TPMi, trabeculated and papillary muscle mass index.

Semiautomatic tracing with manual correction from base to apex was used on end-diastolic and end-systolic SA cine images, and then the threshold-based (TB) algorithm (MassK module of the Medis Suite QMass program) was applied. Based on different signal intensities, the TB program classifies each voxel as blood or myocardium. The voxels identified as myocardium on the end-diastolic images within the epicardial contours represent the total myocardial mass (TM), and those within the endocardial border form the trabeculated and papillary muscle mass (TPM) (Figure 2). The threshold was set to the default of 50% (26). Manual correction of the threshold was not performed.

The LV and RV end-diastolic volume (EDV), end-systolic volume (ESV), stroke volume (SV), and ejection fraction (EF) were also calculated. All parameters were indexed to body surface area (i). As a reference, we used the Alfakih normal values for adults and the percentiles by Kawel-Boehm for children (27, 28).

For the feature-tracking (FT) analysis, commercially available software (QStrain, Medis Suite, version 4.0) was used. The LV endocardial contours were semiautomatically traced with manual correction on the SA view and manually traced on the 2-, 3-, and 4-chamber long-axis views. LV global longitudinal strain (GLS) and global circumferential strain (GCS) were measured and compared with the normal values presented by Peng et al. (29, 30). During the analysis, the strains were interpreted as absolute values: values closer to zero represented worse/poorer function, and values farther from zero represented better function.

Manual correction of the semiautomatically traced epi- and endocardial contours was performed by two observers with 11 years (ASZ) and 7 years (ARK) of experience.

### 2.4 Clinical evaluation and risk stratification

The clinical data, including demographic characteristics, were evaluated by questionnaires at the time of cardiogenetic counseling in the LVNC subjects and at the time of CMR examination in the C group. Detailed family history data included hereditary cardiac diseases or arrhythmia, sudden cardiac death (SCD) or recurrent loss of consciousness, and implanted cardiac devices. Personal medical history included syncope; dizziness; atypical chest pain; palpitation; structural cardiac diseases and arrhythmias; SCD; thromboembolic events; implanted cardiac devices; myocardial infarction; and other comorbidities, such as diabetes mellitus; pulmonary, gastrointestinal, hepatic, kidney, endocrine, oncologic, rheumatic, autoimmune, neurologic, and psychiatric diseases; metabolic syndromes; acute infectious or current cardiac symptoms; and sports activity. LVNC subjects also participated in a personal clinical genetic counseling and genetic testing. Additional information was extracted from previous electronic medical reports.

For a better assessment of the clinical picture, based on the risk stratification recommendation from available literature data, risk factors and negative clinical endpoints were considered “red flags” for clinical evaluation (11–14). These red flags included family history of CMP or SCD in first-degree relatives (subsequently referred to as positive family history), increased LVEDVi on CMR images, ventricular tachycardia or ventricular fibrillation, left bundle branch block (LBBB) or inverted T waves on ECG, medical history of unexplained syncope or thromboembolism or SCD, and nonischemic, mid-myocardial or subepicardial late gadolinium enhancement (LGE) on CMR (11, 12, 14, 19, 27, 28, 31, 32). In previous clinical management, a minor temporary decrease in LVEF (max. to 45%) that normalized after initiation of drug therapy was also considered a red flag in risk stratification (11).

### 2.5 Genetic testing

After genetic counseling, a peripheral venous blood sample was taken from LVNC subjects for genetic testing.

The samples were analyzed using a next-generation sequencing (NGS) method with a 174-gene panel (TruSight Cardio Sequencing Kit, Illumina, CA, USA) containing genes previously associated with cardiac diseases (covering 571,897 nucleotides and 3,251 exons). This method can identify single-nucleotide variations and insertions or deletions in small genetic segments without detecting large CNVs.

During the sequencing reaction, paired-end reads 150 nucleotides in length were synthesized. The *fastq* files converted from optical information were quality controlled using FastQC (v0.11.9) and MultiQC (v1.9) software. The obtained genetic information was compared to the GRCh37.p13 assembly version of reference genome hg19 with BWA software (v0.7.12). Thereafter, to detect mutations, we used Broad Institute GATK software (v4.1.7.0), followed by manual variant screening.

Variant categorization and clinical relevance were determined using the Franklin, NCBI - ClinVar and VarSome databases, with the variants being classified according to the American College of Medical Genetics and Genomics (ACMG) recommendations. To assess previously determined mutation-associated cardiac and extracardiac diseases, we used the ClinGen and OMIM clinical genetic databases.

During genetic data interpretation, pathogenic (P), likely pathogenic (LP), VUS, likely benign and benign mutations were distinguished according to international ACMG guidelines and recommendations (33). In our study, subjects were divided into the following genetic subgroups according to CMP-related mutations: subjects with P and LP variants were combined into the P subgroup, individuals with VUS were classified in the VUS subgroup, and subjects without these mutations were classified in the B subgroup.

Furthermore, CMP-related P and VUS mutations were divided into two subtypes: directly LVNC-related, when the mutation is directly associated with “excessive trabeculation” according to international databases (e.g., Franklin), and other CMP-related, which are mutations that are not directly associated with “excessive trabeculation” but, due to the genetic overlap with CMPs, could have a pathogenic effect (15).

### 2.6 Statistical analyses

Continuous parameters are described as the mean and standard deviation (SD), and discrete parameters are described as numbers and percentages. The homogeneity of variances was assessed with Levene's test, and the normal distribution was assessed with the Shapiro–Wilk test. The differences in baseline and CMR parameters between the LVNC and C groups were assessed with independent t tests when normally distributed and Mann‒Whitney *U*-tests when nonnormally distributed. The continuous parameters among the P, VUS and B subgroups were compared with one-way analysis of variance (ANOVA) and Tukey's *post hoc* test in normally distributed variables with equal variances, Welch test and Games-Howell *post hoc* test in variables with unequal variances, and Kruskal–Wallis test with Bonferroni correction in nonnormally distributed data. The chi-square test and Fisher's exact test were used to compare discrete data. Correlations were assessed with the Pearson correlation coefficient. The multinomial logistic regression model was used to further analyze the connection between red flags and genotype. The interobserver agreement of the two observers was tested using the intraclass correlation coefficient (ICC). A *p*-value <0.05 was considered indicative of statistical significance. IBM SPSS Statistics (Version 28.0) was used for the statistical analyses.

## 3 Results

The interobserver agreement between the two observers was excellent (Supplementary Table S1).

In the genetic classification, 24% (13 subjects) were assigned to the P subgroup, who had relevant CMP-associated likely pathogenic or pathogenic mutations: genes coding TTN were affected in 46%; MYH7, in 15%; and TNNT2, MYBPC3, MIB1, RYR2, SCN5A and KCNQ1, each in 8%. One subject had two different P mutations: TTN and RYR2. Detailed information can be found in Supplementary Table S2A. Fifty-six percent (30 subjects) with VUS in CMP-associated genes were assigned to the VUS subgroup; mutations coding TTN were the most common in this subgroup (29%) (Supplementary Table S2B). All mutations were heterozygous. Twenty percent (11 subjects) without relevant CMP-associated mutations were assigned to the B subgroup.

**Figure 3:**
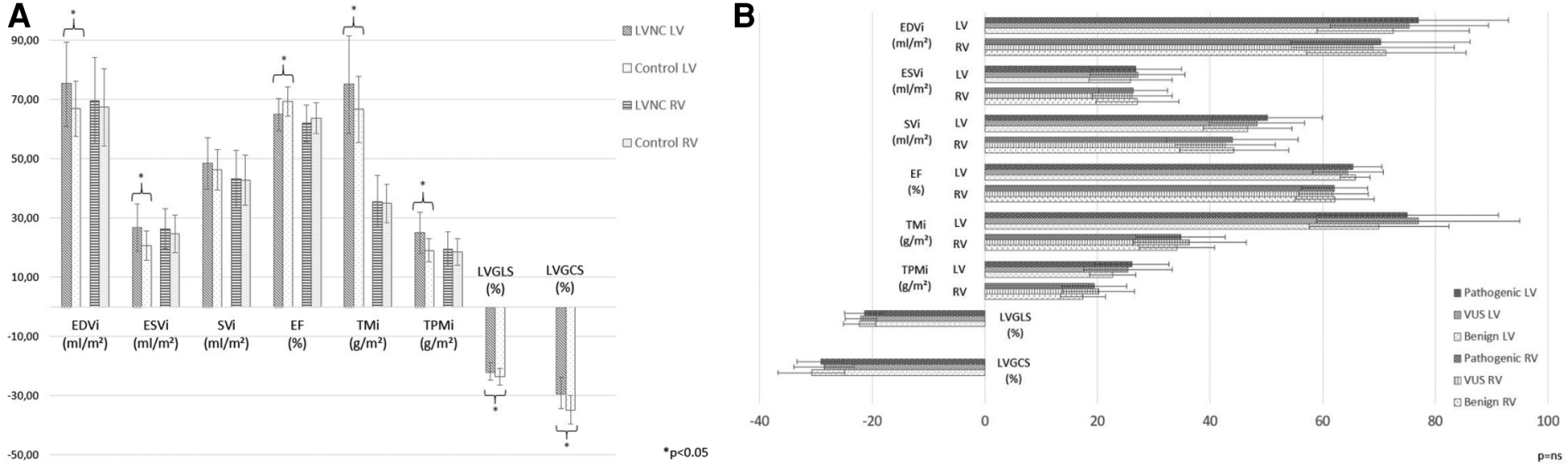
CMR characteristics of the study populations. (**A**) Comparison of LV and RV CMR parameters between the LVNC and C groups. (**B**) Comparison of LV and RV CMR parameters among the three genetic LVNC subgroups. C, control group; CMR, cardiac magnetic resonance imaging; EDVi, end-diastolic volume index; EF, ejection fraction; ESVi, end-systolic volume index; LV, left ventricle; LVGCS, left ventricular global circumferential strain; LVGLS, left ventricular global longitudinal strain; LVNC, left ventricular noncompaction; RV, right ventricle; SVi, stroke volume index; TMi, total muscle mass index; TPMi, trabeculated and papillary muscle mass index; *, *p* < 0.05; ns, nonsignificant.

Comparing the CMR parameters of the LVNC group and the C population, the measured functional parameters were in the normal range, but the LVEDVi, LVESVi, LVTMi and LVTPMi were significantly increased and the LVEF, GLS and GCS values were significantly decreased in the LVNC group. The LVSVi and all the RV parameters did not show significant differences between the two groups (Figure 3A).

The LV and RV functional and strain CMR values were comparable among the three genetic subgroups (Figure 3B), and they did not show significant correlations with genotype (Supplementary Table S3).

Analyzing the clinical symptoms of the LVNC population, a positive family history of CMP or SCD and the recurrence of thromboembolic events were significantly higher in the P and VUS subgroups. SCD and elevated LVEDVi occurred only in the P genotype, and a temporary decrease in LVEF, inverted T wave on ECG and LGE on CMR images were present in both the P and VUS subgroups. Documented arrhythmias, supraventricular arrhythmias, bradycardia and ventricular extrasystole occurred with similar frequency in the three genetic subgroups; ventricular tachycardia and unexplained syncope had a slightly higher prevalence in the P and VUS subgroups; atrioventricular nodal reentry tachycardia (AVNRT) was described in 2 subjects from the VUS subgroup. Despite the minor differences, these abovementioned parameters were statistically comparable. Interestingly, among the subjects' symptoms, the prevalence of atypical chest pain was significantly higher in the B subgroup. Detailed data are reported in Table 2.

**Table 2:** Clinical characteristics of the three LVNC genetic subgroups.

		Genotypes of LVNC population			*p*
		Pathogenic (*n* = 13)	VUS (*n* = 30)	Benign (*n* = 11)	
	Diagnosed in childhood	4	2	1	0.094
	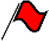 Positive family history	8	15	1	0.024*
Subjects’ symptoms	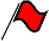 Unexplained syncope	2	2	1	0.816
	Dizziness	6	15	8	0.355
	Atypical chest pain	5	7	8	0.014*
	Palpitations	7	15	6	0.954
Arrhythmia	Nondocumented arrhythmia	3	5	2	0.894
	Documented arrhythmia	5	13	5	0.935
	Supraventricular arrhythmia	3	7	1	0.661
	Atrial fibrillation	0	3	1	0.638
	Ventricular arrhythmia	5	10	5	0.77
	VES	4	9	4	0.925
	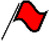 Ventricular tachycardia	2	2	1	0.816
	Bradycardia	1	1	1	0.579
	AVNRT	0	2	0	0.497
Negative endpoints	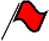 Thromboembolic event	2	0	0	0.038*
	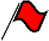 Sudden cardiac death	1	0	0	0.201
ECG signs	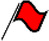 T wave inversion	3	5	0	0.235
	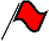 LBBB	1	0	1	0.231
CMR parameters	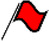 ↑ LVEDVi	2	0	0	0.093
	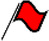 Temporary LVEF ↓	1	4	0	0.698
	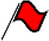 LGE	2	2	0	0.671

AVNRT, atrioventricular nodal reentry tachycardia; CMR, cardiac magnetic resonance imaging; ECG, electrocardiogram; LBBB, left bundle branch block; LGE, late gadolinium enhancement; LVEDVi, left ventricular end-diastolic volume indexed to body surface area; LVEF, left ventricular ejection fraction; LVNC, left ventricular noncompaction; VES, ventricular extrasystole; VUS, variant of uncertain significance; 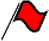, part of the red flag risk stratification model.

* *p* < 0.05.

**Figure 4:**
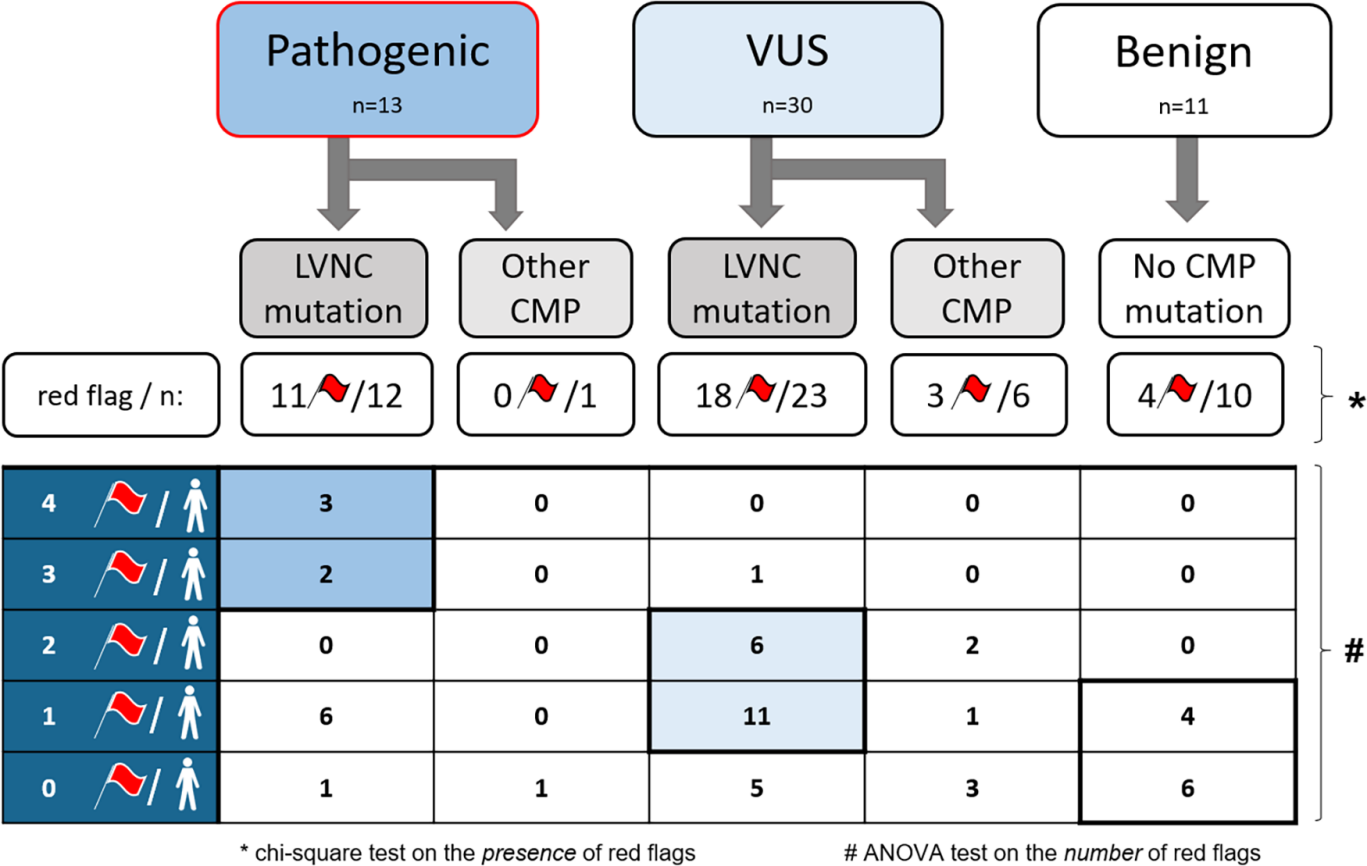
The presence and number of red flags in each genetic subgroup. CMP, cardiomyopathy; LVNC, left ventricular noncompaction; *n*, number of subjects; VUS, variant of uncertain significance; *, *p* < 0.05, the significance level of the chi-square test on the *presence* of red flags among the three genetic subgroups; #, *p* < 0.05, the significance level of the ANOVA test on the *number* of red flags among the three genetic subgroups.

Significant differences were found between the three genetic subgroups regarding the presence of red flags and the number of red flags in a single person (Figure 4). Furthermore, using multinomial logistic regression, the red flag model had a significant association with the genotype (*p* = 0.03), and a moderate positive correlation was found between the number of red flags per person and the genotype (*r* = 0.457, *p* = 0.01).

After these analyses, we divided the P and VUS subgroups into two further genetic subtypes: subjects with at least one directly LVNC-related mutation and subjects with other CMP-related mutations (Figure 4). Remarkably, in the P subgroup, 92% had directly LVNC-related mutations, of whom only one person had no red flags. In the VUS subgroup, we found directly LVNC-related mutations in 77%, 78% of whom had at least one red flag. However, in the B subgroup, 4 subjects (36%) without any mutations had one red flag.

Moreover, the number of red flags in a single person showed significant differences between the directly LVNC-associated and other CMP-related mutations (*p* < 0.001). We also found a significant moderate correlation between the number of red flags per person and the presence of LVNC-associated mutations (*r* = 0.407, *p* = 0.01). Subjects with P LVNC-associated mutations had the largest number of red flags in a single person, which was usually 1–2 in the VUS subgroup, and subjects with the B genotype had no or only 1 red flag per person. Further details are shown in Figure 4.

LVNC was diagnosed in childhood in 7 subjects (13%) of the total LVNC population, and 6 (4 P and 2 VUS) of them had at least one CMP-related P or VUS mutation. Notably, the P subgroup had the highest rate of childhood diagnosis (31%). Detailed information can be seen in Table 2.

## 4 Discussion

In describing the genetic background of a hypertrabeculated LVNC population with preserved LVEF, we also looked for associations among the genotype, imaging characteristics and clinical manifestations that could be easily interpreted by the use of the red flag system.

### 4.1 Relationship between LVNC morphology and genotype

Although the functional CMR parameters of the total LVNC population were within the normal ranges, the LV volumetric and muscle mass parameters were significantly higher, and the LVEF and strain values were significantly lower in the LVNC group than in the C group. These results are consistent with literature data by Zemrak et al. and Kiss et al. (7, 34, 35). In terms of the RV, Kiss et al. reported significantly higher RV volumes and RVTMi in the LVNC group than in controls; however, our results were comparable between the two groups (35). This could be explained by our smaller study population.

In contrast to our results, where the CMR parameters showed no significant differences among the genetic subgroups, previous investigations described the relationship of pathogenic genotype with LV systolic dysfunction and raised a connection with the extent of trabeculation; however, other studies reported no prognostic impact of trabecular mass (7, 11, 19, 21, 36–38). Notably, all these studies were conducted on both preserved and reduced LVEF LVNC populations with a high number of HF patients, and to the best of our knowledge, no data were available about these associations in LVNC subjects with preserved LVEF.

### 4.2 Clinical risk stratification and genetic background

We also intended to analyze the genetic subgroups and their clinical manifestations. Previous studies about the genetic background of LVNC conducted on the heterogeneous spectrum of LVNC populations described similar or slightly higher rates of pathogenic mutations in the same genes as our results, which could be explained by the previously described relationship between LV systolic function and pathogenic genotype (1, 18, 19, 39). Moreover, in patients with severe HF resulting in heart transplantation, this rate was even higher (87.5%) (23). Similarly, according to our results and those of previous studies, the prevalence of P mutations can also be higher in cases of childhood diagnosis (19, 22, 40, 41).

Based on the available literature reviews by Vergani et al. and Negri et al, which emphasize the importance of using red flags in the risk stratification of LVNC subjects and previous studies describing the prognostic effect of clinical characteristics, we developed a new red flag model presented in Methods (11, 12, 14, 19, 27, 28, 31, 32). The novelty of our model is the described statistical association between red flags and genotype. Here, we would like to analyze this revised red flag risk stratification model.

In the literature, major cardiovascular events of LVNC, such as thromboembolic events, arrhythmia and HF symptoms as a part of the red flag system, usually accumulate with systolic dysfunction, which can be associated with the presence of pathogenic genotypes (11, 12, 14, 18, 21, 22). However, complications could occur despite a preserved LVEF, as we found a significantly higher prevalence of thromboembolic events and positive family history (CMP or SCD) in subjects with P and VUS mutations, while SCD occurred in only one case with the P genotype. Interestingly, no previous literature data were found concerning the genotype-phenotype association in the preserved LVEF group.

In addition to the abovementioned clinical entities, the presence of unexplained syncope, ventricular tachycardia or fibrillation, inverted T waves, LBBB, LGE, elevated LVEDVi and temporary decrease in LVEF were also considered parts of this model. Although these issues did not show a significant relationship with P and VUS genetic mutations, probably due to the small sample size, their importance in long-term outcome and risk stratification was described previously (11, 12, 14, 19, 27, 28, 31, 32). Syncope was reported as an early warning sign of ventricular arrhythmias; moreover, some studies revealed the importance of the P genotype in the development of different types of ventricular and supraventricular arrhythmias (12–14, 18, 22, 31, 42). It was also suggested that repolarization abnormalities indicated by inverted T waves and LBBB also have connections with cardiovascular events (12, 43–45). Interestingly, Van Waning et al. reported a higher occurrence of LBBB in sporadic LVNC than in genetically determined LVNC (19). Further red flags, such as elevated LVEDVi and temporarily decreased LVEF, were identified previously as predictors of negative outcomes in LVNC patients (11). Similarly, a recent follow-up study identified LGE as the strongest independent predictor of LVNC-specific complications. Other investigators also described the association between LGE and the P genotype, which is comparable with our results, as we detected LGE only in subjects with CMP-related mutations (11, 32, 36, 37). Interestingly, atypical chest pain was more frequent in subjects without CMP-related mutations, and the prevalence of dizziness and palpitation were comparable among the three genetic subgroups, raising questions regarding their importance in risk stratification.

In contrast to evaluating each clinical parameter separately, some reviews have suggested the importance of a more complex risk stratification as the red flags model, which enables a better characterization of the entire clinical picture. However, no literature data were found concerning its relationship with genotype and its usability in clinical practice (12, 14). Thus, our present study is unique in determining a relationship between genetic mutations and red flags. In this study, we also described not only the presence but also the number of red flags per person: subjects with multiple red flags have a higher probability of P mutations; thus, in these cases, genetic testing can be recommended. In contrast, LVNC subjects with no or only one red flag have a lower possibility of CMP-related mutations, so they might require a more flexible follow-up during clinical management (1). These results are supported by the “Expert Consensus Statement on the state of genetic testing for cardiac diseases” by Wilde et al., in which similar clinical circumstances are considered indicators of the need for genetic testing (46). Interestingly, we found significantly more red flags in subjects with directly LVNC-related mutations than in individuals with other CMP-related mutations. This may support specific aspects of diagnosing the “excessive trabeculation” phenotype and its follow-up management.

Finally, the importance of the VUS subgroup in the genetic classification must be mentioned, as in our study, subjects with these mutations usually had one or two red flags per person. According to the ACMG guidelines, VUS mutations should not be considered in clinical decision-making, but additional monitoring may be necessary during the follow-up of these subjects, which is also underlined by our results (2, 33). Furthermore, the interpretation of VUS can be difficult in daily medical practice, as new clinical data can modify the current ACMG database.

## 5 Conclusion

In our study, we highlighted on the genetic background of symptomatic LVNC subjects with preserved LVEF, and detected P genotype in one-quarter of the studied population. Analyzing the genetic subgroups, despite the comparable phenotype based on CMR parameters, higher number of red flags were observed among subjects with genetic mutations. As new recommendations suggest the importance of clinical symptoms and etiology in the hypertrabeculated phenotype, our study proved that the use of a red flag system might help to support risk stratification and clinical management; however, more follow-up data are required.

## 6 Limitations

Although this cross-sectional genetic study was performed on a large LVNC population, the follow-up was limited and the sample size of the genetic subgroups may have affected the statistical findings.

Another limitation of our study could be that the 174-gene panel possibly does not cover all variants, and the continually refreshing clinical data may also modify the international ACMG databases, leading to the potential reclassification of VUS.

Finally, it should be noted that the TB software uses SA images with an 8 mm standard for spatial resolution in the Z-direction for the quantification of cardiac volumes and EF. Due to the fact that trabeculae and papillary muscles do not cross the slice exactly perpendicularly, the actual path of the trabeculae may affect the TB measurements.

## 7 Scope statement

In this study, we characterized the genetic, imaging and clinical background of a symptomatic hypertrabeculated or left ventricular noncompaction (LVNC) phenotype population with preserved ejection fraction (LVEF), compared them to a healthy control group and investigated the genotype-phenotype relationships. Twenty-four percent of our population carried pathogenic mutations in cardiomyopathy-associated genes, and 56% of subjects had variants of uncertain significance (VUS). Despite the comparable phenotype on CMR images among subjects with different genotypes, differences in clinical manifestation and family history were noted. Symptomatic hypertrabeculated subjects with genetic involvement had a higher number of risk factors, identified as “red flags” in our study, than subjects without genetic mutations; and were more likely to be diagnosed in childhood.

Although several articles focusing on LVNC with heart failure describe high rates of genetic involvement, the genotype of symptomatic hypertrabeculation with preserved LVEF and its effect on the clinical presentation have been less studied in the literature. Thus, our study suggests new concepts about the genetic and clinical characteristics of symptomatic hypertrabeculation with preserved LVEF: the large number of pathogenic mutations and the association between red flags and genotype underline the importance of genetic-assisted risk stratification in symptomatic LVNC with preserved LVEF.

## Data Availability

The original contributions presented in the study are included in the article/Supplementary Material, further inquiries can be directed to the corresponding author.
