# Supplementary material for: Genetic, clinical and imaging implications of a noncompaction phenotype population with preserved ejection fraction
Source: Front Cardiovasc Med. 2024 Feb 6;11:1337378. doi: 10.3389/fcvm.2024.1337378 (PMC10876896; doi:10.3389/fcvm.2024.1337378)
Supplement: Supplementary file 1 [file Table1.docx]

**Supplementary Table 1:** Interobserver agreement (ICC) for the measured left and right ventricular functional and strain parameters between the two observers

|  | **Intraclass correlation coefficient** |
| --- | --- |
| **LVEDVi** | 0.965 (0.860-0.991) |
| **LVESVi** | 0.972 (0.885-0.993) |
| **LVSVi** | 0.943 (0.770-0.986) |
| **LVEF** | 0.895 (0.576-0.974) |
| **LVTMi** | 0.993 (0.970-0.998) |
| **LVTPMi** | 0.979 (0.914-0.995) |
| **RVEDVi** | 0.991 (0.965-0.998) |
| **RVESVi** | 0.955 (0.818-0.989) |
| **RVSVi** | 0.995 (0.979-0.999) |
| **RVEF** | 0.943 (0.769-0.986) |
| **RVTMi** | 0.988 (0.952-0.997) |
| **RVTPMi** | 0.986 (0.945-0.997) |
| **LVGLS** | 0.956 (0.889-0.983 |
| **LVGCS** | 0.955 (0.886-0.982) |

ICC = intraclass correlation: average measures (95% confidential interval lower and upper band), LVEDVi = left ventricular end-diastolic volume index, LVESVi = left ventricular end-systolic volume index, LVSVi = left ventricular stroke volume index, LVEF = left ventricular ejection fraction, LVTMi = left ventricular total mass index, LVTPMi = left ventricular trabeculated and papillary muscle mass index, RVEDVi = right ventricular end-diastolic volume index, RVESVi = right ventricular end-systolic volume index, RVSVi = right ventricular stroke volume index, RVEF = right ventricular ejection fraction, RVTMi = right ventricular total mass index, RVTPMi = right ventricular trabeculated and papillary muscle mass index, LVGLS = left ventricular global longitudinal strain, LVGCS = left ventricular global circumferential strain

**Supplementary Table 2:** The identified CMP-related mutations in our LVNC study population

**Supplementary Table 2A:** Identified pathogenic and likely pathogenic mutations in CMP-related genes in our LVNC study population

|  | **Gene symbol** | **Affected protein** | **variant_ID** | **Transcript ID** | **HGVSc** | **HGVSp** |
| --- | --- | --- | --- | --- | --- | --- |
| **LVNC–related mutations** | **TTN** | Titin | chr2-179659682-G-C | ENST00000589042 | c.1212C>G | p.Tyr404Ter |
|  |  |  | chr2-179536824-C-A | ENST00000589042 | c.35101G>T | p.Glu11701Ter |
|  |  |  | chr2-179427059-AG-A | ENST00000589042 | c.83799del | p.Phe27934SerfsTer3 |
|  |  |  | chr2-179392275-G-A | ENST00000589042 | c.107578C>T | p.Gln35860Ter |
|  |  |  | chr2-179397981-TC-T | ENST00000589042 | c.103360del | p.Glu34454AsnfsTer3 |
|  |  |  | chr2-179604901-TG-T | ENST00000589042 | c.13058del | p.Pro4353GlnfsTer14 |
|  | **MYH7** | Myosin Heavy Chain 7 | chr14-23901869-C-T | ENST00000355349 | c.481G>A | p.Ala161Thr |
|  |  |  | chr14-23897873-T-C | ENST00000355349 | c.1414A>G | p.Ser472Gly |
|  | **TNNT2** | Troponin T2, Cardiac Type | chr1-201333463-CG-C | ENST00000509001 | c.421del | p.Arg141GlyfsTer41 |
|  | **MYBPC3** | Myosin Binding Protein C3 | chr11-47353740-G-A | ENST00000545968 | c.3697C>T | p.Gln1233Ter |
|  | **MIB1** | MIB E3 Ubiquitin Protein Ligase 1 | chr18-19426998-C-T | ENST00000261537 | c.2305C>T | p.Arg769Ter |
|  | **RYR2** | Ryanodine Receptor 2 | chr1-237947092-C-T | ENST00000366574 | c.12080C>T | p.Thr4027Met |
| **Other CMP-related mutations** | **SCN5A** | Sodium Voltage-Gated Channel Alpha Subunit 5 | chr3-38597188-G-C | ENST00000413689 | c.4501C>G | p.Leu1501Val |
|  | **KCNQ1** | Potassium Voltage-Gated Channel Subfamily Q Member 1 | chr11-2799221-G-A | ENST00000155840 | c.1748G>A | p.Arg583His |

**Supplementary Table 2B:** Identified VUS in CMP-related genes in our LVNC study population

| **LVNC–related mutations** | **Gene symbol** | **Affected protein** | **variant_ID** | **Transcript ID** | **HGVSc** | **HGVSp** |
| --- | --- | --- | --- | --- | --- | --- |
|  | **TTN** | Titin | chr2-179440214-C-T | ENST00000589042 | c.70645G>A | p.Val23549Ile |
|  |  |  | chr2-179396214-C-T | ENST00000589042 | c.105128G>A | p.Arg35043His |
|  |  |  | chr2-179497044-C-T | ENST00000589042 | c.43577G>A | p.Arg14526Gln |
|  |  |  | chr2-179638835-G-A | ENST00000589042 | c.7060C>T | p.Arg2354Cys |
|  |  |  | chr2-179664608-T-C | ENST00000589042 | c.613A>G | p.Lys205Glu |
|  |  |  | chr2-179590352-A-T | ENST00000589042 | c.20579T>A | p.Leu6860Gln |
|  |  |  | chr2-179395825-CAGA-C | ENST00000589042 | c.105514_105516del | p.Ser35172del |
|  |  |  | chr2-179434420-C-T | ENST00000589042 | c.76439G>A | p.Arg25480His |
|  |  |  | chr2-179441038-C-T | ENST00000589042 | c.69821G>A | p.Gly23274Asp |
|  |  |  | chr2-179480082-C-T | ENST00000589042 | c.48590G>A | p.Arg16197His |
|  |  |  | chr2-179485269-G-A | ENST00000589042 | c.45979C>T | p.Arg15327Cys |
|  |  |  | chr2-179454443-C-A | ENST00000589042 | c.62009G>T | p.Gly20670Val |
|  |  |  | chr2-179606268-A-C | ENST00000589042 | c.11692T>G | p.Tyr3898Asp |
|  |  |  | chr2-179411491-C-T | ENST00000589042 | c.94664G>A | p.Arg31555His |
|  |  |  | chr2-179470215-C-T | ENST00000589042 | c.53807G>A | p.Arg17936His |
|  |  |  | chr2-179593289-T-A | ENST00000589042 | c.19364A>T | p.Tyr6455Phe |
|  |  |  | chr2-179395924-C-T | ENST00000356239 | c.8245A>G | p.Ile2749Val |
|  |  |  | chr2-179401778-A-G | ENST00000589042 | c.100058T>C | p.Ile33353Thr |
|  |  |  | chr2-179397619-G-A | ENST00000589042 | c.103723C>T | p.Arg34575Cys |
|  |  |  | chr2-179428543-T-C | ENST00000589042 | c.82316A>G | p.Glu27439Gly |
| **LVNC–related mutations** |  |  | chr2-179399941-C-G | ENST00000589042 | c.101401G>C | p.Glu33801Gln |
|  |  |  | chr2-179462290-C-A | ENST00000589042 | c.57519G>T | p.Lys19173Asn |
|  |  |  | chr2-179440696-C-T | ENST00000589042 | c.70163G>A | p.Arg23388Gln |
|  |  |  | chr2-179428168-G-A | ENST00000589042 | c.82691C>T | p.Ala27564Val |
|  |  |  | chr2-179473599-T-A | ENST00000589042 | c.52139A>T | p.Asp17380Val |
|  | **MYH6** | Myosin Heavy Chain 6 | chr14-23859571-G-A | ENST00000405093 | c.3427C>T | p.Arg1143Trp |
|  | **MYH7** | Myosin Heavy Chain 7 | chr14-23885311-C-T | ENST00000355349 | c.4855G>A | p.Glu1619Lys |
|  |  |  | chr14-23889090-G-T | ENST00000355349 | c.3690C>A | p.Asp1230Glu |
|  | **MYPN** | Myopalladin | chr10-69948821-C-T | ENST00000358913 | c.2863C>T | p.Arg955Trp |
|  |  |  | chr10-69881632-C-G | ENST00000358913 | c.437C>G | p.Ser146Cys |
|  | **ACTC1** | Actin Alpha Cardiac Muscle 1 | chr15-35083407-C-T | ENST00000290378 | c.898G>A | p.Val300Ile |
|  | **PKP2** | Plakophilin 2 | chr12-33021917-C-G | ENST00000070846 | c.1114G>C | p.Ala372Pro |
|  |  |  | chr12-33003744-A-G | ENST00000070846 | c.1334T>C | p.Val445Ala |
|  | **LDB3** | LIM Domain Binding 3 | chr10-88451678-G-A | ENST00000429277 | c.919G>A | p.Val307Ile |
|  | **DSP** | Desmoplakin | chr6-7565642-T-G | ENST00000379802 | c.828T>G | p.Ile276Met |
|  | **LMNA** | Lamin A/C | chr1-156085004-C-A | ENST00000368300 | c.295C>A | p.Arg99Ser |
|  | **PRDM16** | PR/SET Domain 16 | chr1-3347507-  GGAGGAGGACGACGATGACCT-A | ENST00000270722 | c.3369_3389del | p.Asp1124_Asp1130del |
|  |  |  | chr1-3103007-C-T | ENST00000270722 | c.356C>T | p.Ala119Val |
|  |  |  | chr1-3342777 G>A | ENST00000270722 | c.3272G>A | p.Arg1091Gln |
|  | **TBX20** | T-Box Transcription Factor 20 | chr7-35288360-CTTG-C | ENST00000408931 | c.471_473del | p.Asn157del |
|  | **SEPN1** | Selenoprotein N | chr1-26138338-C-T | ENST00000361547 | c.1249C>T | p.Arg417Cys |
|  | **RYR2** | Ryanodine Receptor 2 | chr1-237837399 G>A | ENST00000366574 | c.8594G>A | p.Gly2865Glu |
|  |  |  | chr1-237947183-C-G | ENST00000366574 | c.12171C>G | p.His4057Gln |
|  |  |  | chr1-237758826-T-C | ENST00000366574 | c.4465T>C | p.Cys1489Arg |
|  | **DTNA** | Dystrobrevin Alpha | chr18-32428343-C-T | ENST00000598334 | c.1169C>T | p.Ser390Leu |
| **Other CMP-related mutations** | **MYO6** | Myosin VI | chr6-76564978-G-A | ENST00000369977 | c.1201G>A | p.Gly401Ser |
|  | **MYOZ2** | Myozenin 2 | chr4-120085436-A-T | ENST00000307128 | c.447A>T | p.Gln149His |
|  | **ACTN2** | Actinin Alpha 2 | chr1-236883470-A-C | ENST00000366578 | c.427A>C | p.Ile143Leu |
|  | **PDLIM3** | PDZ And LIM Domain 3 | chr4-186444529-T-G | ENST00000284770 | c.317A>C | p.Glu106Ala |
|  |  |  | chr4-186423579-C-T | ENST00000284770 | c.964G>A | p.Asp322Asn |
|  | **JUP** | Junction Plakoglobin | chr17-39912027-G-A | ENST00000393931 | c.2207C>T | p.Pro736Leu |
|  | **NEXN** | Nexilin F-Actin Binding Protein | chr1-78383933-G-A | ENST00000334785 | c.422G>A | p.Arg141His |
|  | **ANKRD1** | Ankyrin Repeat Domain 1 | chr10-92679936-C-T | ENST00000371697 | c.197G>A | p.Arg66Gln |
|  |  |  | chr10-92679936-C-T | ENST00000371697 | c.197G>A | p.Arg66Gln |
|  | **TRIM63** | Tripartite Motif Containing 63 | chr1-26380423-C-A | ENST00000374272 | c.1012G>T | p.Asp338Tyr |
|  |  |  | chr1-26384907-C-T | ENST00000374272 | c.805G>A | p.Glu269Lys |
|  |  |  | chr1-26384907-C-T | ENST00000374272 | c.805G>A | p.Glu269Lys |
|  |  |  | chr1-26384907-C-T | ENST00000374272 | c.805G>A | p.Glu269Lys |
|  | **BAG3** | BAG Cochaperone 3 | chr10-121431767-C-T | ENST00000369085 | c.508C>T | p.Arg170Trp |
|  |  |  | chr10-121431767-C-T | ENST00000369085 | c.508C>T | p.Arg170Trp |
|  | **ABCC9** | ATP Binding Cassette Subfamily C Member 9 | chr12-22025560-T-C | ENST00000261200 | c.2197A>G | p.Asn733Asp |
|  | **DSG2** | Desmoglein 2 | chr18-29121188-G-A | ENST00000261590 | c.1912G>A | p.Gly638Arg |
|  |  |  | chr18-29104840-A-G | ENST00000261590 | c.1003A>G | p.Thr335Ala |
|  |  |  | chr18-29121188-G-A | ENST00000261590 | c.1912G>A | p.Gly638Arg |
|  | **GLA** | Galactosidase Alpha | chrX-100653420-C-A | ENST00000218516 | c.937G>T | p.Asp313Tyr |
|  | **TNNI3** | Troponin I3, Cardiac Type | chr19-55666150-T-C | ENST00000344887 | c.331A>G | p.Arg111Gly |
|  | **TGFB3** | Transforming Growth Factor Beta 3 | chr14-76427292-G-A | ENST00000238682 | c.1054C>T | p.Arg352Cys |

CMP = cardiomyopathy, LVNC = left ventricular noncompaction, VUS = variant of uncertain significance, ID = identifier, HGVSc = human genome variation society coding DNA sequence, HGVSp = human genome variation society protein sequence

**Supplementary Table 3:** The correlation between genotype and CMR parameters in our LVNC study population

| **Correlation between genotype and CMR parameters** | **r** | **p** |
| --- | --- | --- |
| **LVEDVi (ml/m^2^)** | 0.104 | 0.454 |
| **LVESVi (ml/m^2^)** | 0.038 | 0.783 |
| **LVSVi (ml/m^2^)** | 0.136 | 0.328 |
| **LVEF (%)** | -0.018 | 0.896 |
| **LVTMi (g/m^2^)** | 0.095 | 0.496 |
| **LVTPMi (g/m^2^)** | 0.161 | 0.243 |
| **LVGLS (%)** | 0.099 | 0.477 |
| **LVGCS (%)** | 0.097 | 0.483 |
| **RVEDVi (ml/m^2^)** | -0.020 | 0.888 |
| **RVESVi (ml/m^2^)** | -0.032 | 0.820 |
| **RVSVi (ml/m^2^)** | -0.007 | 0.960 |
| **RVEF (%)** | -0.003 | 0.980 |
| **RVTMi (g/m^2^)** | 0.019 | 0.892 |
| **RVTPMi (g/m^2^)** | 0.103 | 0.458 |

CMR = cardiac magnetic resonance imaging, LVEDVi = left ventricular end-diastolic volume index, LVESVi = left ventricular end-systolic volume index, LVSVi = left ventricular stroke volume index, LVEF = left ventricular ejection fraction, LVTMi = left ventricular total mass index, LVTPMi = left ventricular trabeculated and papillary muscle mass index, RVEDVi = right ventricular end-diastolic volume index, RVESVi = right ventricular end-systolic volume index, RVSVi = right ventricular stroke volume index, RVEF = right ventricular ejection fraction, RVTMi = right ventricular total mass index, RVTPMi = right ventricular trabeculated and papillary muscle mass index, LVGLS = left ventricular global longitudinal strain, LVGCS = left ventricular global circumferential strain, r = Pearson correlation coefficient, p = significance level of the correlation
